# Prediction of Attendance Demand in European Football Games: Comparison of ANFIS, Fuzzy Logic, and ANN

**DOI:** 10.1155/2018/5714872

**Published:** 2018-08-07

**Authors:** Mehmet Şahin, Rızvan Erol

**Affiliations:** ^1^Department of Business Administration, Adiyaman University, 02040 Adiyaman, Turkey; ^2^Department of Industrial Engineering, Cukurova University, 01330 Adana, Turkey

## Abstract

An artificial neural network (ANN), adaptive neurofuzzy inference system (ANFIS) models, and fuzzy rule-based system (FRBS) models are developed to predict the attendance demand in European football games, in this paper. To determine the most successful method, each of the methods is analyzed under different situations. The Elman backpropagation, feed-forward backpropagation, and cascade-forward backpropagation network types are developed to determine the outperforming ANN model. The backpropagation and hybrid optimization methods are used for training fuzzy inference system (FIS) to determine the outperforming ANFIS model. The fuzzy logic model is developed after experimenting different forms of membership functions. To this end, the data of 236 soccer games are used to train the ANN and ANFIS models, and 2017/2018 season's data of these clubs are used to test all of the models. The results of all models are compared with each other and real past data. To assess the performance of each model, two error measures that are Mean Absolute Percent Error (MAPE) and Mean Absolute Deviation (MAD) are implemented. These measures reveal that the ANN model that has Elman network type outperforms the other models. Finally, the results emphasize that the proposed ANN model can be effectively used for prediction purposes.

## 1. Introduction

In recent years, the economic impact of sports events has grown significantly [1]. Thus, the attendance demand has become a prominent topic in sports economics and marketing literature [2]. Demand information is utilized by decision makers in sports such as regulators, executives, and administrators [3]. Demand information is one of the substantial inputs for planning as well. Therefore, accurate demand forecasting plays a central role in the economics of clubs.

The usual approach used in previous studies in the sports literature is based on predicting a linear demand equation. For a detailed literature review, readers can refer to Borland and MacDonald [3] and Villar and Guerrero [4]. The first econometric analysis study of soccer games is presented by Hart et al. [5]. Pawlowski and Anders [6] analyzed German Bundesliga matches and find that the attendance is related to the possibility of winning the championship. Reilly [7] examined the Ireland football league and found that the uncertainty of outcome, quality of schedule, performance of teams, and rivalry are effective factors. Martins and Cró [1] analyzed five seasons of the Portuguese First Division League, and found that weekend and derby games attract more spectators. Dobson and Goddard [8] examined English football clubs and found that the population of hometown, league membership period of a team, and the competition degree are effective determinants on the spectator attendance. Serrano et al. [9] examined the relationship between the quality of the event and demand at European football stadiums. García and Rodríguez [2] examined games in the Spanish First Division Football League and found that the team quality is the most important factor among the factors. Dubin [10] analyzed the data of National Football League (NFL) and found that team performance has significant effects on demand, but it is not the only contributing factor. Lemke et al. [11] examined Major League Baseball (MLB) games and found that attendance increases as the chance of the home team winning the game increases.

The central aim of this study is to evaluate the performance of three alternative forecasting techniques that are NN, ANFIS, and fuzzy logic and reveal the most accurate forecasting technique for predicting attendances of European football games. For this purpose, real data of soccer games are used. By using past real data, the attendances of soccer games are forecasted by each of the methods. The attendance rates of soccer games were predicted depending on five effective variables that are the day of the game, the distance in terms of miles between stadia of home and away clubs, uncertainty of outcome, and the home and away teams' performances. These factors are determined after examining the literature in detail and interviewing with experts. The uncertainty of outcome as a determining factor is included in this study since it covers the effect of significant factors such as injured players, suspended players, and so on. Each of the models was tested comprehensively under different scenarios. The forecasting results of these three methods were compared to each other and past real data. The proposed models are not limited to forecasting demand of European football games as they can be utilized in a variety sports disciplines by making some alterations.

Fuzzy logic is a computation and reasoning system where the objects of computation and reasoning are classes with fuzzy boundaries. In fuzzy logic, everything is to be a matter of degree [12]. Fuzzy logic system allows analysis for complicated system structures in which for modeling the system, linguistic expressions are used rather than numerical variables. Fuzzy logic has been increasingly used in different fields such as economics [13], image processing, power engineering, systems engineering, optimization, industrial automation, and robotics with a variety purposes [14]. Some of the studies in sports are as follows: Novatchkov and Baca [15] designed a fuzzy logic model for the assessment of strength training exercises in sports. Martínez et al. [16] proposed a fuzzy logic approach for measuring perceived quality in terms of sport and fitness services. Şahin and Erol [17] designed a fuzzy logic model for forecasting demand of soccer games. Chua et al. [18] designed a fuzzy logic approach for pool games that is an intelligent decision system.

Neural networks simulate the functioning of the biologic neurons. NN has the ability to learn from experiences and information to enhance its performance [19]. NN has been implemented to different problems in various areas. After the neurological network models by McCulloch and Pitts [20], a great number of neural network models have been designed. NN studies in sports concentrate on selecting talents and players and predicting the winner of a game, results of games, and success of teams. McCullagh [21] designed an NN approach for player selection in the annual draft of Australian Football League (AFL) and found that the NN model might be utilized for the talent identification to assist recruiting managers. Maszczyk et al. [22] used NNs in the javelin throwers' selection and found that NN models are capable for assisting in the recruiting process of javelin throwers. Huang and Chang [23] used 2006 World Cup data and designed an NN model to estimate the winning rate of the game for home and away teams and achieved 76.9% prediction accuracy. Purucker [24] analyzed the use of NNs to estimate the winning team in the NFL. Rotshtein et al. [25] used neural networks, fuzzy logic, and genetic algorithm to predict football game results. Loeffelholz et al. [26] designed an NN model to predict the teams' success in the National Basketball Association (NBA) and achieved a prediction accuracy of 74.33%. Condon et al. [27] designed linear regression and NN models to estimate the achievement of participating nations in the Summer Olympic Games and found that the results of the best NN model are better than the best regression model. Strnad et al. [28] designed an NN model to forecast the attendance of soccer matches and found that NN provided better forecasts than the traditional regression model. Şahin and Erol [29] designed an NN model to forecast attendances of soccer games and found that the model provides effective results.

ANFIS was first introduced by Jang [30]. The ANFIS combines the advantages of NN and Fuzzy Inference Systems (FIS). Thus, it has rapid learning capacity, the capability of seizing the nonlinear structure of a process, capability of adaptation, and is not requiring expert knowledge. The ANFIS has been successfully implemented to a broad range of problems in a variety of fields including economics [31], transportation [32], energy [33], health [34] and the environment [35] for different purposes including diagnosis [34], evaluation [36], prediction [37], and forecasting [38]. However, it is in infancy in sports in which it has been used for simulated soccer agents [39] and forecasting attendance of soccer games recently [29].

This study extends the literature in the following ways. First, differently from the previous study [29], an effective input variable, uncertainty of outcome, is included in the ANFIS and ANN models. Since the uncertainty of outcome covers the effect of significant factors such as injured and suspended players, the accuracy of attendance prediction is improved. Second, Elman and cascade-forward backpropagation networks are designed for the first time for that purpose to see whether the types of networks affect the accuracy of the prediction. Nine ANN models are designed, and the performance of each is evaluated. Third, two ANFIS models are designed to reveal the outperforming one. Fourth, differently from the previous study [17], the performance of away team factor is included in the fuzzy logic model. Additionally, a large data set is used for testing the models. Last but not least, this is the first study to compare the three of the effective soft computing techniques for this purpose.

## 2. Materials and Methods

### 2.1. Data

Data are required to train and validate the ANN and ANFIS models and test all of the models including the fuzzy logic. Thus, the attendance data of a Spanish football club, FC Barcelona, and two Italian football clubs, AC Milan and FC Inter, are obtained. The data of 236 games of the three clubs are used to predict the attendance demand rates of their 2017–2018 season games. Data sources of the input variables are given in Table 1.

The data are standardized to obtain better forecasting results. This is fulfilled by employing maximum linear standardization whose formula is given as follows [40]:

(1) $$\begin{array}{c} X_{i}=\frac{X_{\text{o}i}}{\max\;\;X_{\text{o}i}}, \end{array}$$

where *X*_*i*_ represents value *i* of the input data after the standardization and *X*_o*i*_ represents value *i* of the input data before the standardization. By using the data, the ANFIS, NN, and fuzzy logic models are designed to predict attendance rates in European football games. Since each football club has a different sized stadium capacity, the attendances differ from club to club. Therefore, as a general term, the attendance rate (occupancy rate) is preferred as the output in this study.

### 2.2. Methods

#### 2.2.1. Fuzzy Logic

**Figure 1:**
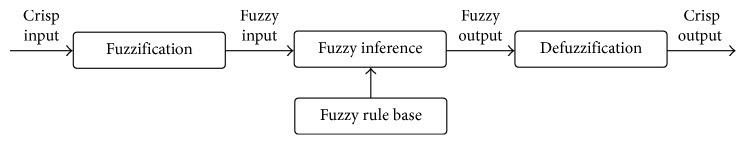
Fuzzy logic system.

Fuzzy logic is a modeling technique in which two human capabilities, which are the reasoning ability and the ability to fulfill different mental tasks, are tried to be mechanized [41]. In fuzzy logic systems, linguistic expressions are used instead of numerical variables for modeling. Fuzzy modeling is fundamentally a rule-based system that is stated as fuzzy inference system as well. A typical fuzzy logic system consists of four major parts that are fuzzification, fuzzy rule base, fuzzy inference, and defuzzification as is illustrated in the Figure 1.

To design a fuzzy rule-based model, the following steps are essential. First, the input and output variables are determined. Second, the fuzzy sets are determined for all variables. Third, the membership functions of all fuzzy inputs and outputs are created. There are different kinds of membership functions such as triangular, Gaussian, trapezoidal, and so on. Since type of membership functions impact the design of the fuzzy logic controller, they should be chosen carefully. Fourth, the fuzzy IF-THEN rules are generated to relate input and output variables. Fifth, the inference process is set. The two most common FIS types are the Sugeno and Mamdani. There are some differences between them. The output of the Sugeno is linear or constant, but the output of Mamdani comprises of membership functions that may be trapezoidal, triangular, and so on. Additionally, Sugeno is trained using data set, but Mamdani does not require a data set and relies on expert knowledge. In this study, the Mamdani-type fuzzy inference system is preferred in the rule-based fuzzy logic model by utilizing the expert knowledge. The Mamdani type comprises of the following processes. The input variables are fuzzified so that to the degree they fit to each of the fuzzy set is established over membership functions. Next, an “AND” or “OR” fuzzy operator is used to combine the inputs to provide a single number. Next, the rule's weight is set before the implication that is implemented for each rule. Next, all of the fuzzy rules are combined and evaluated. The outputs are aggregated by the aggregation methods including max (maximum), probor (probabilistic OR), and sum (simply the sum of each rule's output set). Thus, the outputs of each rule are combined into a fuzzy set that need defuzzification [42]. The sixth step is the defuzzification in which the fuzzy results are converted to crisp output values. Some of the defuzzification methods are mean of maximum (MOM), centroid, smallest of maximum (SOM), and largest of maximum (LOM).

#### 2.2.2. Adaptive Neurofuzzy Inference System

The ANFIS can solve any kind of complex and nonlinear problems effectively by combining the advantages of the NN and fuzzy logic. It combines numerical and linguistic knowledge by utilizing fuzzy methods. It also uses the ANN's ability of data classification and pattern identification. Additionally, the ANFIS causes less memorization errors and is more observable to the user compared to the ANN.

The ANFIS is fundamentally the rule-based fuzzy modeling. Fuzzy rules are formed through the training process [43]. The training is performed by using a data set. The ANFIS designs a fuzzy inference system (FIS) and the parameters of membership functions are formed based on the training data. In the ANFIS model, the Sugeno-type FIS is utilized as the data set is used.

**Figure 2:**
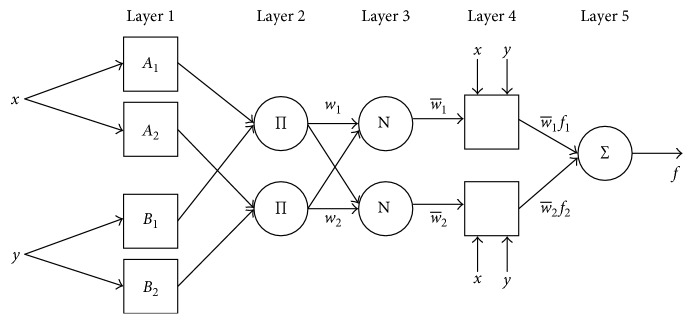
The architecture of the ANFIS model with two inputs, one output, and two rules.

To describe the architecture of ANFIS, which is shown in Figure 2, *x* and *y* are considered to be inputs. A Sugeno-type fuzzy model with two fuzzy IF-THEN rules is represented as follows:

(2) $$\begin{array}{c} \text{Rule}\;\;1:\;\;\mathrm{If}\;\;x\;\;\mathrm{is}\;\;A_{1}\;\;\mathrm{and}\;\;y\;\;\mathrm{is}\;\;B_{1},\;\text{then}\;\;f_{1}=p_{1}x+q_{1}y+r_{1},\\ \text{Rule}\;\;2:\;\;\mathrm{If}\;\;x\;\;\mathrm{is}\;\;A_{2}\;\;\mathrm{and}\;\;y\;\;\mathrm{is}\;\;B_{2},\;\text{then}\;\;f_{2}=p_{2}x+q_{2}y+r_{2}, \end{array}$$

where *A*_*i*_ and *B*_*i*_ stand for the fuzzy sets, *f*_*i*_ stands for the output, and *p*_*i*_, *q*_*i*_, and *r*_*i*_ stand for the design parameters that are set during the process of training.

The ANFIS architecture has five layers that can be described as follows, where *O*_*i*_^*j*^ represents the output of the node *i* and layer *j* [44]:

(i) In the layer 1, every node is defined by the function as
  (3) $$\begin{array}{c} O_{i}^{1}=\mu_{A_{i}}\left(x\right),\;\text{for}\;\;i=1,2, \end{array}$$
  where *x* stands for the input node *i* and *A*_*i*_ stands for the linguistic label.
(ii) In the layer 2, every node calculates the firing strength of a rule by multiplication:
  (4) $$\begin{array}{c} O_{i}^{2}=w_{i}=\mu_{A_{i}}\left(x\right)\;*\;\mu_{B_{i}}\left(y\right),\;i=1,2. \end{array}$$
(iii) In the layer 3, evaluated firing strengths are normalized:
  (5) $$\begin{array}{c} O_{i}^{3}={\bar{w}}_{i}=\frac{w_{i}}{w_{1}+w_{2}},\;i=1,2. \end{array}$$
(iv) In the layer 4, node *i* computes the addition of rule *i* to the output:
  (6) $$\begin{array}{c} O_{i}^{4}={\bar{w}}_{i}\;*\;f_{i}={\bar{w}}_{i}\left(p_{i}\;*\;x+q_{i}\;*\;y+r_{i}\right), \end{array}$$
  where ${\bar{w}}_{i}$ is the output of layer 3 and the parameter set is {*p*_*i*_, *q*_*i*_, *r*_*i*_}.
(v) In the layer 5, the single node computes the overall output of the ANFIS:
  (7) $$\begin{array}{c} O_{1}^{5}=\underset{i}{\sum }{\bar{w}}_{i}\;*\;f_{i}=\frac{\sum_{i}w_{i}\;*\;f_{i}}{\sum_{i}w_{i}}. \end{array}$$

The ANFIS consists of backpropagation and hybrid learning algorithms that focus on minimizing the error between the observed and forecasted data [45]. In this study, both of them are applied to compare the results of them.

#### 2.2.3. Neural Network

The neural network, which is one of the Artificial Intelligence (AI) techniques, can be defined as a computational tool whose processing is similar to the behavior of biological neurons. In other words, the NN may be described as a mathematical demonstration of the individuals' neural architecture [46]. It is trained by using data, so restrictive assumptions are not mandatory in the designing process of the model. After training the NN, it has ability to respond to new data. The NN detects complex nonlinear structures between dependent and independent variables [47]. It can be applied to the problems in which the relationship between input and output is complex or uncertain.

**Figure 3:**
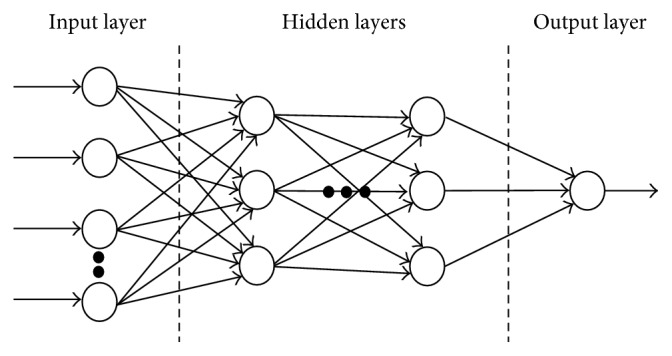
Multilayer Perceptron (MLP) network architecture.

By depending on the disposition of neurons and the composition of the layers, the architectures of the ANN is classified as recurrent NN, single-layer feed-forward NN, and multilayer feed-forward NN [48]. Multilayer Perceptron (MLP), whose general structure is shown in Figure 3, uses multilayer feed-forward architecture and is the most commonly applied network. A MLP comprises of three layers that are input, hidden, and output. The input layer receives features of input data and distributes them to the hidden layer. The hidden layer contains neurons, and it transforms the input to the form that the output layer is able to interpret. The output layer contains neurons as well and produces the final outputs.

The optimal NN structure is formed after trial and errors in general [49]. After all possible NN structures are trained and tested by using the data set, the structure of the NN model, which provides the best results that provides the smallest error, is chosen. The NN is trained over again until the anticipated accuracy level has been achieved. The neural networks are divided into two groups as supervised and unsupervised networks. The unsupervised networks (i.e., competitive layers and self-organizing maps) are trained by allowing the network continuously adjust itself to new inputs. The supervised networks are trained by using data to generate needed outputs regarding inputs. Supervised networks are generally appropriate for modeling and controlling dynamic systems, classifying data, and forecasting. feed-forward networks (feed-forward backpropagation, cascade-forward backpropagation, perceptron, etc.), radial basis networks (generalized regression and probabilistic NNs), and dynamic networks (Elman, Hopfield, nonlinear autoregressive, etc.) are the supervised networks. For the scope of this study, Elman, feed-forward backpropagation, and cascade-forward backpropagation networks are used. There are various training functions that are selected based on the size and type of a problem. Levenberg–Marquardt is one of the fastest and most efficient training functions, and it is appropriate for training small and medium-sized networks [50]. Therefore, it is chosen in this study.

### 2.3. Model Evaluation

The performance of forecasting results of each model is evaluated using the MAPE and MAD, which are calculated by the following formulae:

(8) $$\begin{array}{c} \text{MAPE}=\frac{1}{n}\overset{n}{\underset{t=1}{\sum }}\left|\frac{A_{t}-F_{t}}{A_{t}}\right|, \end{array}$$

where *F*_*t*_ stands for the expected value for period *t*, *A*_*t*_ stands for the actual value for period *t*, and *n* stands for the total period number. The result of the MAPE explains accuracy as an error percentage.

(9) $$\begin{array}{c} \text{MAD}=\frac{1}{n}\overset{n}{\underset{t=1}{\sum }}\left|F_{t}-A_{t}\right|, \end{array}$$

For both statistical indicators, MAPE and MAD, smaller values usually indicate more effective results. In this study, the MAPE and MAD values for each model are obtained by comparing the predicted results with the real past data.

### 2.4. Application of the Models

In order to design effective forecasting models, the selection of input variables is one of the fundamental issues in the modeling system. The input variables should be chosen in a way that the model relates input and output variables effectively and provides accurate results. To predict attendances of European football games, five input variables have been identified by evaluating the literature thoroughly and expert knowledge. Considering the characteristics of European football games, the following effective factors are chosen. The first one is the ground distance between the home and away teams' stadia [3, 8, 51, 52]. This factor reflects the negative effect of long distances, and the positive effect of local derby effect to attendances. In this study, the ground distance is considered. The second one is the day of the game [53, 54]. Weekend games attract more spectators than those on weekdays in general [2]. In this study, the days are numbered from 1 to 7 sequentially. The third one is the performance of home team. This factor is considered to be more effective compared to the performance of away teams by Rascher [55] and Bruggink and Eaton [45] for the MLB games and by Forrest and Simmons [52] for soccer games. The positive performance of the home team attracts more spectators [52]. The performance of the home team is calculated, as in the study by Forrest and Simmons [52], by dividing points are earned by the team by possible total points to the date of the game. The fourth one is the performance of away team [5, 52]. Highly placed away teams attracted more spectators [5]. The performance of away team is calculated similarly to the performance of home team. The last one is the uncertainty of outcome. This factor is defined as the unpredictability degree in terms of the score of a game by Forrest and Simmons [52] who posit that the attendance decreases as uncertainty decreases. Fans prefer more an even league over a less balanced one [56]. To measure this factor, the betting odds are utilized as Peel and Thomas [51] and Forrest and Simmons [52] state in their research. They posit that odds are set by assessing all of the other factors, which are injured players, suspended players, and so on, affecting attendance of the game. In this study, the smaller odd is divided by the larger one as spectators prefer even games that are expected to be more exciting. All of the five input variables are used in the ANN, ANFIS, and fuzzy logic models that are designed in MATLAB R2017a (Mathworks, MA, USA).

**Figure 4:**
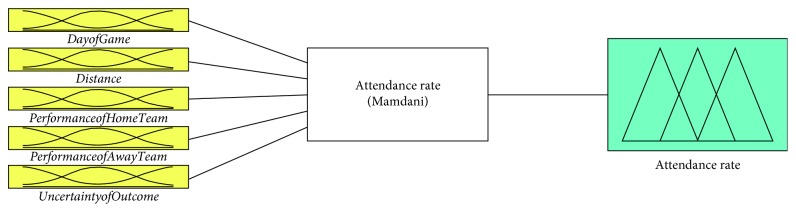
The structure of the proposed fuzzy rule-based model.

The structure of the developed fuzzy rule-based model is illustrated in Figure 4. In the fuzzy logic model, the fuzzy sets are defined for input variables and the output variable as given in Table 2.

**Figure 5:**
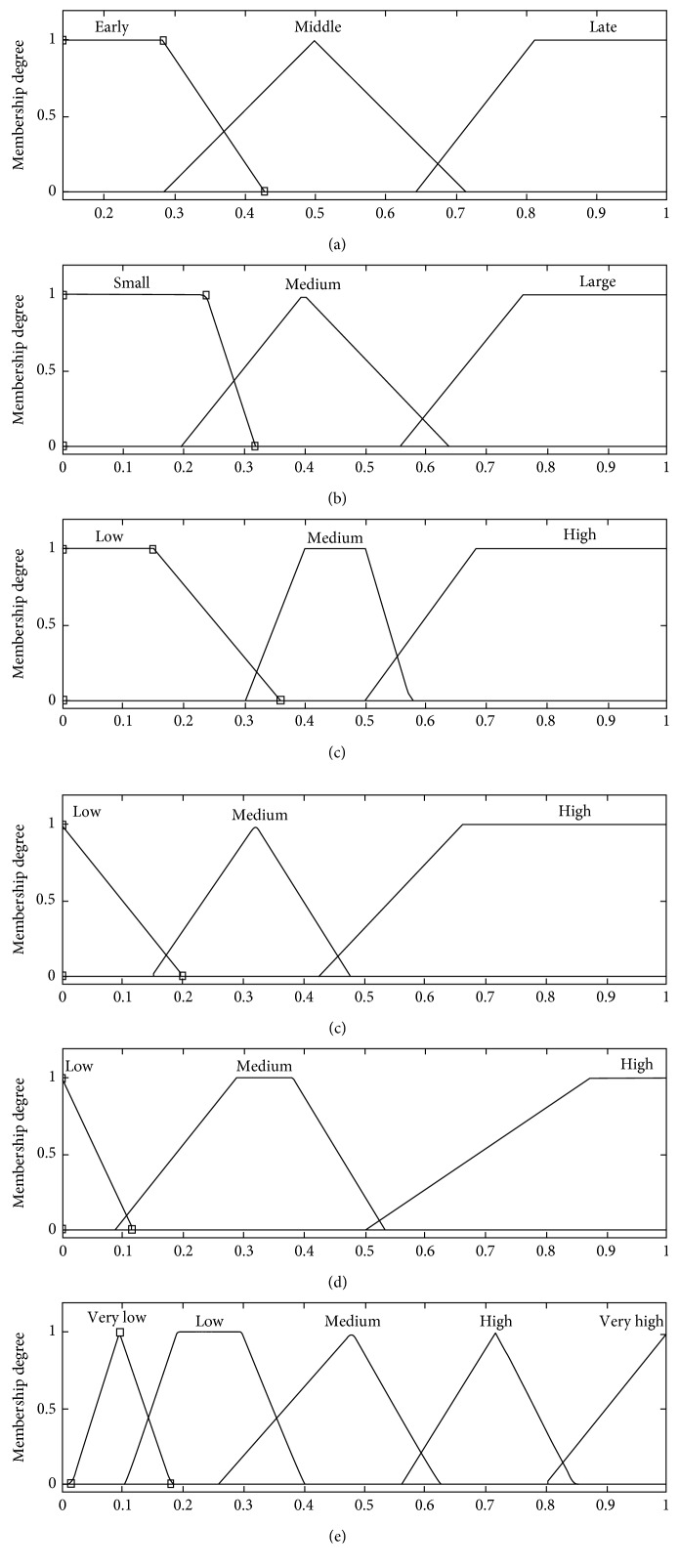
Membership functions formed for the input variables (a) day of game, (b) distance, (c) performance of home team, (d) performance of away team, (e) uncertainty of outcome, and the output variable (f) attendance rate.

Next, the membership functions of all variables are formed. The most appropriate type is generally chosen after experimenting different types. The membership functions of the proposed model are shown in Figure 5. The ranges of these functions are set depending on the regarding studies. For instance, Dobson and Goddard [8] consider distances of up to 60 miles as small and distances of greater than 200 miles as large. These values are normalized as mentioned before, so the values on the figure are the normalized ones. Next, 32 fuzzy IF-THEN rules are generated depending on the thorough literature review and expert knowledge. One of them is given as follows:

If (*DayofGame is not Early*) and (*Distance is not Large*) and (*PerformanceofHomeTeam is High*) and (*PerformanceofAwayTeam is High*) and (*UncertaintyofOutcome is High*) then (*AttendanceRate is VeryHigh*)

As seen in the fuzzy rule, the five conditions are related to each other with AND operators. As a FIS, the Mamdani-type inference system is chosen as explained before. Finally, to obtain crisp values, the centroid method that takes the center of the area under the curve is chosen as the defuzzification method.

**Figure 6:**
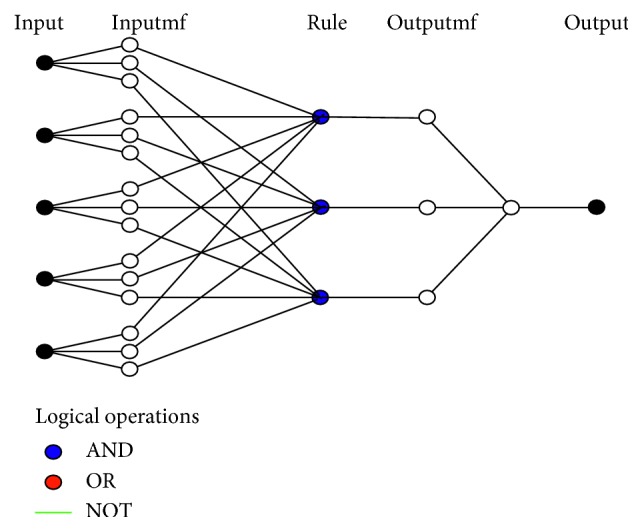
The structure of the proposed ANFIS models.

In the ANFIS models, the subtractive clustering is chosen to generate FIS since the prediction accuracy obtained is higher compared to the grid partitioning. Three membership functions are formed for each input variable. Two different optimization methods, which are hybrid and backpropagation, are used for training FIS. Thus, different number of epochs, which are 100 and 1000, respectively, is established for accurate prediction results. The parameters for subtractive clustering and features of the ANFIS models are given in Table 3. These parameters are determined based on the accuracy of the prediction results. As it can be seen in table, there are three membership functions for each input variable. The structure of the developed ANFIS model is shown in Figure 6.

**Figure 7:**
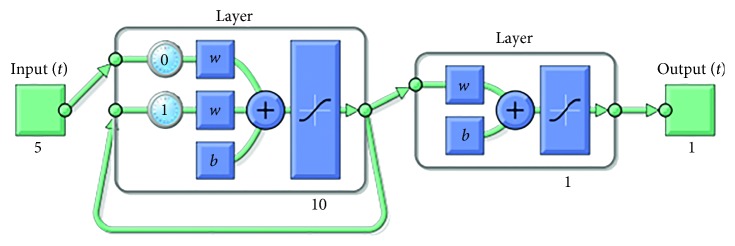
The proposed one-layer Elman NN model (10 neurons).

**Figure 8:**
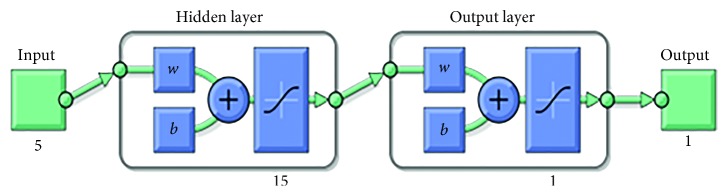
The proposed one-layer feed-forward NN model (15 neurons).

**Figure 9:**
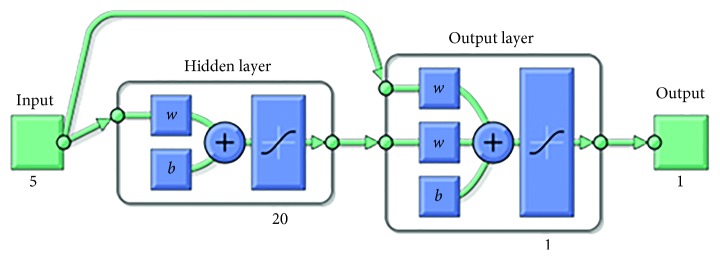
The proposed one-layer cascade-forward NN model (20 neurons).

In the ANN models, three different network types, which are Elman backpropagation, feed-forward backpropagation, and cascade-forward backpropagation, are used to determine the outperforming one. For all network types, one hidden layer is designed since the number of inputs, five, is not high. The designed Elman, feed-forward, and cascade-forward backpropagation ANN models are shown in Figures 7–9, respectively. Different numbers of neurons are chosen for each network type to evaluate performances of them. 10, 15, and 20 neurons are chosen for each network type, respectively. Nine models are designed in total. The properties of the models are given in Table 4.

## 3. Results and Discussion

The proposed models are designed and implemented in MATLAB R2017a. The observed data are calculated by dividing the number of spectators attended to the game by the stadium capacity.

### 3.1. Fuzzy Logic

The proposed fuzzy logic model provides the following predicted attendance rates as shown in Table 5. In the table, observed attendance rates are compared to the predicted attendance rates, and differences between them are shown. In general, difference values indicate that the predicted rates seem effective. Especially, the prediction result of the game 12 seems to be the most accurate. However, the accuracy level of the prediction results of the games 5, 6, 7, 15, and 21 appear to be low. The predictions are high for the games 6, 7, and 21. Even though the performance of AS Roma is high, the attendances of its games are lower than the expected. There may be another factor that causes attendances to be low. In addition, for the game 6, the effect of the game day might not be reflected to the predictions enough. However, for the games 5 and 15, the attendances appear to be underestimated. For the game 5, there may be special case other than these factors since the performance of Girona FC and uncertainty of outcome are not high. In addition, for the game 15, the big game effect might be ignored even though the performance of away team and uncertainty of outcome were selected to reflect that effect.

To evaluate the performance of the model, the following MAPE and MAD indicators are computed as given in Table 6. As it is seen from the table, the MAPE value is equal to 0.1 and the MAD value is 0.07 that is fewer than 10%. It can be concluded that the model provides effective and competitive predictions overall.

### 3.2. ANFIS

As mentioned before, two ANFIS models are designed. The prediction results of the models are shown in Table 7. In the table, the predicted attendance rates by each model are shown. The differences between predicted attendance rates and the observed attendance rate are also given. The first model uses hybrid optimization method for training FIS. The prediction accuracy of this model is high overall. However, the estimates for games 3 and 9 seem inaccurate since they are overestimated. The performances of away teams were high relatively. Therefore, the model might consider them as highly demanded games. The second model uses backpropagation optimization method for training FIS. The prediction accuracy of this model is low compared to the first model. The prediction results for the games 2, 5, 6, 9, 10, 12, and 16 appear to be inaccurate. The most inaccurate predictions, games 2 and 9, might be a result of ignoring the effect of performance of away team. However, it provided one of the most accurate results for the games 15 and 19.

To evaluate the performance of both models, the MAPE and MAD values are obtained compared to the observed data as shown in Table 8. These results of the statistical indicators show that the ANFIS model using hybrid optimization method provides more effective predictions than the ANFIS model using the backpropagation. Even though the measures indicate that both models appear to be effective, by looking at the prediction results in detail, the more appropriate model between two is the ANFIS using hybrid method for this purpose.

### 3.3. ANN

Nine ANN models are designed in total. Three different network types, in which each has three different numbers of neurons, provide different attendance predictions as shown in Table 9. The first model, whose network type is Elman backpropagation, is tested under three different situations that are with 10, 15, and 20 neurons. The second model, whose network type is feed-forward backpropagation, is tested under three different situations that are with 10, 15, and 20 neurons either. The third model, whose network type is cascade-forward backpropagation, is tested under three different situations that are with 10, 15, and 20 neurons as well. To evaluate the prediction results of the models, the differences between the results of the proposed models, and the actual data are given in Table 10. As it can be observed in the table, the most inaccurate estimates are provided by the feed-forward backpropagation network type that has 1 hidden layer with 20 neurons. Despite its accurate predictions for games 4, 6, 7, 8, 13, 15, 16, 17, and 19, the deviation of its predictions are high for other games. Other than that, the most accurate predictions are provided by the Elman network type that has 1 hidden layer with 20 neurons. By looking at the differences between this model and the observed data, the most ineffective predictions are provided for games 10 and 21. Still, these predictions are acceptable compared to the results of the other models. This model provides the most accurate result for the game 16.

By looking at Table 10, the following results can be inferred. The Elman network type that has 1 hidden layer with 10 neurons provides the most accurate prediction for game 12; the Elman with 15 neurons for games 6, 9, and 20. The feed-forward network type that has 1 hidden layer with 10 neurons provides the most accurate prediction for game 2; the feed-forward with 15 neurons for games 5, 11, and 17; the feed-forward with 20 neurons for games 4, 14, and 15. The cascade network type that has 1 hidden layer with 10 neurons provides the most accurate predictions for games 1, 8, 10, 18, 19, 21, and 22; the cascade with 15 neurons for games 7; the cascade with 20 neurons for games 3, 13, and 23. Hence, the cascade network type that has 1 hidden layer with 10 neurons is also competitive and effective for this purpose due to its the most accurate prediction results for seven games in total.

To determine the most accurate ANN model, the MAPE and MAD values for all models are obtained as given in Table 11. As it is seen from the table, the error measures are generally under 10% that shows that the models provide accurate predictions overall. To determine the outperforming model, all MAPE and MAD values are compared. Thus, it can be concluded that the Elman network type that has 1 hidden layer with 20 neurons provides the most accurate predictions among the nine models. In addition, the feed-forward backpropagation network type that has 1 hidden layer with 20 neurons provides the most inaccurate estimates among all models.

### 3.4. Comparison of the ANFIS, ANN, and Fuzzy Logic

In this section, the outperforming models of ANN, ANFIS, and fuzzy logic approaches are compared with the observed data and each other as shown in Table 12. The Elman network type that has 1 hidden layer with 20 neurons provides the most effective predictions for games 6, 11, 16, and 22. The ANFIS, which uses hybrid optimization method for training FIS, provides the most accurate estimates for games 1, 5, 7, 14, 15, 17, 19, 21, and 23. The proposed fuzzy logic model delivers the most effective predictions for the rest ten games.

**Figure 10:**
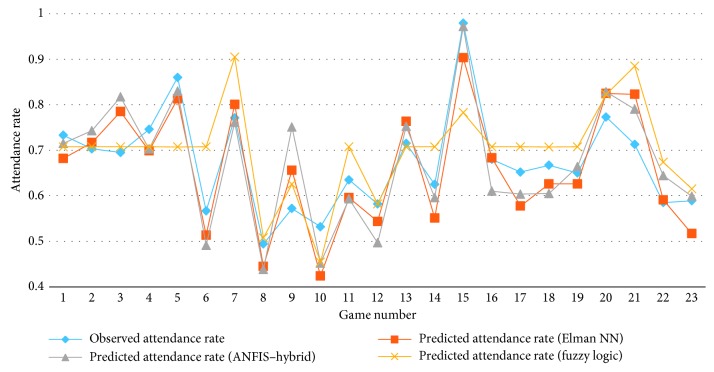
The comparison of the outperforming ANFIS, ANN, and fuzzy logic models with the observed data.

The comparisons of the prediction game-by-game are shown in Figure 10. This figure illustrates that the predicted attendance rates are close to each other and the observed attendance rates. This demonstrates the success of all of the proposed models.

To determine the outperforming model among twelve prediction models, MAPE and MAD values of the best models of each technique are compared as shown in Table 13.

As it is seen from the table and explained before, three techniques provide accurate and effective predictions. However, the Elman network type that has one hidden layer with 20 neurons is the most successful and outperforming for this purpose. However, the performance of the proposed ANFIS model is not that bad. By adding data from different clubs from different countries and training the ANFIS model, its performance might be improved. In addition, the performance of the proposed fuzzy logic model might be enhanced as well by making few modifications. The fuzzy rules might be modified.

## 4. Conclusions

In this study, nine ANN models, two ANFIS models, and a fuzzy logic model are designed to predict attendance demand in European football games. Since results of demand forecasting are crucial inputs for decision making and planning, the accuracy of the forecasting is vital. Therefore, the most effective attendance determinants are chosen after a comprehensive literature review and interviewing with experts. The distance, game day, performance of the home team, performance of the away team, and uncertainty of outcome are selected and used in all twelve models in this study.

The 236 games' data of three European football clubs are utilized for training the ANN and ANFIS models, and one season's data of the club are used for testing the ANN, ANFIS, and fuzzy logic models. The performance of each model is evaluated by two statistical indicators that are MAPE and MAD. Based on the prediction results, it can be inferred that the ANN, ANFIS, and fuzzy logic provide effective and competitive predictions since the MAPE and MAD values are generally under 10%. However, the ANN model whose network type is the Elman that has one hidden layer with 20 neurons delivers the most accurate and effective results among twelve models. The MAPE and MAD values of the model are 0.08 and 0.05, respectively, meaning that the prediction accuracy is high in general as well.

Even though the NN, ANFIS, and fuzzy logic models were proposed with the similar purpose before, this study extended the literature by the following additions. First, including the uncertainty of outcome that covers the effective factors in ANN and ANFIS models improved the accuracy of the predictions. Second, a large, diverse data set is used to train and test the models, and different input variables are established in the fuzzy logic model. Finally, nine ANN models are designed that allow a comprehensive analysis of the network types. Thus, the Elman network type that provides the most effective prediction results among ANN models is proposed for the first time in this study.

Future research may analyze different network types of the ANN technique. In addition, alternative effective demand factors may be included in the models to evaluate whether prediction results are improved or not. Finally, a larger data set may be used to train models.

## Figures and Tables

**Table 1:** Data sources of input variables.

Input variable	Data source
Performance of the home team	The official websites of home teams
Day of the game	The official websites of home teams
Performance of away teams	The official websites of away teams
Distance	http://www.distancecalculator.net
Uncertainty of outcome	http://www.football-data.co.uk

**Table 2:** Fuzzy sets of the variables.

Fuzzy sets of input variables					Fuzzy sets of output variable
Day of game	Distance (miles)	Performance of home team	Performance of away team	Uncertainty of outcome	Attendance rate
Early	Small	Low	Low	Low	Very low
Middle	Medium	Medium	Medium	Medium	Low
Late	Large	High	High	High	Medium
—	—	—	—	—	High
—	—	—	—	—	Very high

**Table 3:** The properties of the proposed ANFIS models.

Parameter	Description/value
Type of fuzzy inference system	Sugeno
Optimization method for training FIS	Subtractive clustering
Range of influence	0.87
Squash factor	1.1
Accept ratio	0.5
Reject ratio	0.1
Input number	5
Output number	1
Number of input membership functions	3, 3, 3, 3, 3
Optimization methods	Hybrid; backpropagation
Training epoch numbers	100; 1000

**Table 4:** The features of the ANN models.

Network type	Elman	Feed-forward	Cascade-forward
Number of layers	2	2	2
Input	5	5	5
Neurons	Hidden: 10; 15; 20	Hidden: 10; 15; 20	Hidden: 10; 15; 20
Output	1	1	1
Training algorithm	Levenberg–Marquardt	Levenberg–Marquardt	Levenberg–Marquardt

**Table 5:** The observed and predicted attendance rates of the proposed fuzzy logic model.

Number	Game	Observed attendance rate	Predicted attendance rate	Difference
1	Barcelona-Espanyol	0.733	0.7076	0.026
2	Barcelona-Malaga	0.703	0.7076	−0.004
3	Barcelona-Sevilla	0.695	0.7075	−0.012
4	Barcelona-Getafe	0.746	0.7075	0.038
5	Barcelona-Girona	0.860	0.7073	0.152
6	Milan-SPAL	0.567	0.7076	−0.141
7	Milan-Roma	0.771	0.9046	−0.134
8	Milan-Bologna	0.494	0.5076	−0.014
9	Milan-Atalanta	0.572	0.6255	−0.054
10	Milan-Crotone	0.532	0.4545	0.077
11	Milan-Lazio	0.635	0.7075	−0.072
12	Milan-Sampdoria	0.582	0.5821	0.000
13	Inter-SPAL	0.715	0.7076	0.008
14	Inter-Genoa	0.625	0.7076	−0.082
15	Inter-Milan	0.979	0.7827	0.196
16	Inter-Sampdoria	0.680	0.7076	−0.027
17	Inter-Atalanta	0.652	0.7075	−0.055
18	Inter-Chievo	0.667	0.7072	−0.040
19	Inter-Udinese	0.650	0.7076	−0.058
20	Inter-Lazio	0.773	0.8226	−0.050
21	Inter-Roma	0.713	0.8847	−0.172
22	Inter-Crotone	0.585	0.6737	−0.089
23	Inter-Benevento	0.589	0.6153	−0.026

**Table 6:** MAPE and MAD values of the proposed fuzzy logic model.

Error measures	Fuzzy logic
MAD	0.07
MAPE	0.1

**Table 7:** Predicted attendance rates of the ANFIS models.

Number	ANFIS–hybrid		ANFIS–backpropagation	
	Predicted attendance rate	Difference	Predicted attendance rate	Difference
1	0.7153	0.018	0.6534	0.080
2	0.7431	−0.040	0.8420	−0.139
3	0.8172	−0.122	0.7728	−0.078
4	0.7029	0.043	0.7108	0.035
5	0.8300	0.030	0.7590	0.101
6	0.4912	0.075	0.4628	0.104
7	0.7612	0.010	0.7188	0.052
8	0.4381	0.056	0.4377	0.056
9	0.7510	−0.179	0.7506	−0.179
10	0.4522	0.079	0.4211	0.110
11	0.5932	0.042	0.5678	0.067
12	0.4967	0.086	0.4756	0.107
13	0.7521	−0.037	0.6733	0.042
14	0.5960	0.029	0.5958	0.030
15	0.9718	0.007	0.9815	−0.003
16	0.6102	0.070	0.5702	0.110
17	0.6037	0.048	0.5730	0.079
18	0.6050	0.062	0.6022	0.065
19	0.6638	−0.014	0.6453	0.005
20	0.8284	−0.055	0.8537	−0.081
21	0.7902	−0.077	0.7295	−0.016
22	0.6445	−0.060	0.5682	0.017
23	0.5989	−0.010	0.5328	0.057

**Table 8:** MAPE and MAD values of the proposed ANFIS models.

Error measures	ANFIS–hybrid	ANFIS–backpropagation
MAD	0.05	0.07
MAPE	0.09	0.11

**Table 9:** Predicted attendance rates of the ANN models.

Number	Predicted attendance rates								
	Elman (10 n.)	Elman (15 n.)	Elman (20 n.)	Feed-forward (10 n.)	Feed-forward (15 n.)	Feed-forward (20 n.)	Cascade (10 n.)	Cascade (15 n.)	Cascade (20 n.)
1	0.6824	0.8157	0.6821	0.6965	0.6513	0.5026	0.7222	0.8148	0.8153
2	0.7167	0.7558	0.7168	0.7055	0.6342	0.8690	0.8293	0.5956	0.7328
3	0.8484	0.7915	0.7848	0.7986	0.7488	0.8274	0.8096	0.7770	0.7009
4	0.7577	0.7347	0.6988	0.7218	0.7064	0.7379	0.7127	0.6668	0.6234
5	0.7748	0.7924	0.8128	0.7732	0.8198	0.7810	0.7802	0.8027	0.6234
6	0.5293	0.5795	0.5135	0.5800	0.5484	0.5966	0.5373	0.6586	0.7041
7	0.8783	0.8702	0.8009	0.7958	0.8384	0.8027	0.7532	0.7873	0.6618
8	0.4377	0.4396	0.4449	0.4500	0.4514	0.4454	0.4530	0.4381	0.4478
9	0.6335	0.5802	0.6561	0.5224	0.7232	0.8754	0.7309	0.6917	0.6388
10	0.4167	0.4521	0.4239	0.4689	0.4874	0.9415	0.5034	0.4456	0.4907
11	0.7226	0.6883	0.5958	0.5237	0.6519	0.3890	0.5491	0.7369	0.4813
12	0.5552	0.5135	0.5436	0.5367	0.4743	0.4676	0.5075	0.5539	0.5352
13	0.8922	0.8611	0.7636	0.8545	0.8776	0.6948	0.8228	0.7760	0.6995
14	0.6605	0.6632	0.5511	0.7128	0.5111	0.6442	0.7148	0.7101	0.6906
15	0.8709	0.9132	0.9031	0.9593	0.9143	0.9723	0.8558	0.8923	0.9336
16	0.6431	0.6420	0.6833	0.8884	0.7360	0.6439	0.7098	0.8056	0.8008
17	0.7518	0.7169	0.5774	0.5762	0.6571	0.6943	0.6172	0.5982	0.6691
18	0.7603	0.7430	0.6260	0.6906	0.7074	0.5893	0.6552	0.6540	0.5848
19	0.7046	0.6786	0.6261	0.6920	0.7004	0.6014	0.6402	0.6698	0.5929
20	0.8080	0.7675	0.8248	0.7449	0.8608	0.9138	0.7650	0.8357	0.8793
21	0.8424	0.8067	0.8229	0.8493	0.8167	0.9099	0.7388	0.8230	0.7678
22	0.6025	0.6041	0.5912	0.5570	0.6711	0.8681	0.5898	0.6774	0.6387
23	0.5210	0.5235	0.5170	0.5642	0.5030	0.6915	0.5251	0.5185	0.5831

**Table 10:** Differences between the predicted and observed attendance rates.

Number	Differences between observed and predicted attendance rates								
	Elman (10 n.)	Elman (15 n.)	Elman (20 n.)	Feed-forward (10 n.)	Feed-forward (15 n.)	Feed-forward (20 n.)	Cascade (10 n.)	Cascade (15 n.)	Cascade (20 n.)
1	0.051	−0.082	0.051	0.037	0.082	0.231	0.011	−0.081	−0.082
2	−0.013	−0.053	−0.014	−0.002	0.069	−0.166	−0.126	0.108	−0.030
3	−0.153	−0.096	−0.090	−0.103	−0.054	−0.132	−0.114	−0.082	−0.006
4	−0.012	0.011	0.047	0.024	0.039	0.008	0.033	0.079	0.122
5	0.085	0.067	0.047	0.087	0.040	0.079	0.079	0.057	0.236
6	0.037	−0.013	0.053	−0.013	0.018	−0.030	0.029	−0.092	−0.137
7	−0.107	−0.099	−0.030	−0.025	−0.067	−0.032	0.018	−0.016	0.109
8	0.056	0.054	0.049	0.044	0.042	0.048	0.041	0.056	0.046
9	−0.062	−0.009	−0.084	0.049	−0.152	−0.304	−0.159	−0.120	−0.067
10	0.115	0.079	0.108	0.063	0.044	−0.410	0.028	0.086	0.041
11	−0.087	−0.053	0.039	0.111	−0.017	0.246	0.086	−0.102	0.154
12	0.027	0.069	0.039	0.046	0.108	0.115	0.075	0.028	0.047
13	−0.177	−0.146	−0.048	−0.139	−0.162	0.020	−0.107	−0.061	0.016
14	−0.035	−0.038	0.074	−0.087	0.114	−0.019	−0.089	−0.085	−0.065
15	0.108	0.066	0.076	0.020	0.065	0.007	0.123	0.087	0.045
16	0.037	0.038	−0.003	−0.208	−0.055	0.037	−0.029	−0.125	−0.120
17	−0.100	−0.065	0.075	0.076	−0.005	−0.042	0.035	0.054	−0.017
18	−0.093	−0.076	0.041	−0.023	−0.040	0.078	0.012	0.013	0.083
19	−0.055	−0.029	0.024	−0.042	−0.050	0.049	0.010	−0.020	0.057
20	−0.035	0.006	−0.052	0.028	−0.088	−0.141	0.008	−0.063	−0.106
21	−0.129	−0.093	−0.110	−0.136	−0.104	−0.197	−0.026	−0.110	−0.055
22	−0.018	−0.019	−0.006	0.028	−0.086	−0.283	−0.005	−0.092	−0.054
23	0.068	0.066	0.072	0.025	0.086	−0.102	0.064	0.071	0.006

**Table 11:** MAPE and MAD values of the proposed ANN models.

Error measures	Elman (10 n.)	Elman (15 n.)	Elman (20 n.)	Feed-forward (10 n.)	Feed-forward (15 n.)	Feed-forward (20 n.)	Cascade (10 n.)	Cascade (15 n.)	Cascade (20 n.)
MAD	0.07	0.06	0.05	0.06	0.07	0.12	0.06	0.07	0.07
MAPE	0.11	0.09	0.08	0.09	0.10	0.19	0.09	0.11	0.11

**Table 12:** The comparison of the predictions of the proposed models.

Game	Observed attendance rate	Predicted attendance rate (Elman NN–20 n.)	Predicted attendance rate (ANFIS–hybrid)	Predicted attendance rate (fuzzy logic)
Barcelona-Espanyol	0.733	0.6821	0.7153	0.7076
Barcelona-Malaga	0.703	0.7168	0.7431	0.7076
Barcelona-Sevilla	0.695	0.7848	0.8172	0.7075
Barcelona-Getafe	0.746	0.6988	0.7029	0.7075
Barcelona-Girona	0.860	0.8128	0.8300	0.7073
Milan-SPAL	0.567	0.5135	0.4912	0.7076
Milan-Roma	0.771	0.8009	0.7612	0.9046
Milan-Bologna	0.494	0.4449	0.4381	0.5076
Milan-Atalanta	0.572	0.6561	0.7510	0.6255
Milan-Crotone	0.532	0.4239	0.4522	0.4545
Milan-Lazio	0.635	0.5958	0.5932	0.7075
Milan-Sampdoria	0.582	0.5436	0.4967	0.5821
Inter-SPAL	0.715	0.7636	0.7521	0.7076
Inter-Genoa	0.625	0.5511	0.5960	0.7076
Inter-Milan	0.979	0.9031	0.9718	0.7827
Inter-Sampdoria	0.680	0.6833	0.6102	0.7076
Inter-Atalanta	0.652	0.5774	0.6037	0.7075
Inter-Chievo	0.667	0.6260	0.6050	0.7072
Inter-Udinese	0.650	0.6261	0.6638	0.7076
Inter-Lazio	0.773	0.8248	0.8284	0.8226
Inter-Roma	0.713	0.8229	0.7902	0.8847
Inter-Crotone	0.585	0.5912	0.6445	0.6737
Inter-Benevento	0.589	0.5170	0.5989	0.6153

**Table 13:** Comparison of the MAPE and MAD values of the proposed models.

Error measures	ANN–Elman–20 neurons	ANFIS–hybrid	Fuzzy logic
MAD	0.05	0.05	0.07
MAPE	0.08	0.09	0.1

## Data Availability

The data used to support the findings of this study are available from the corresponding author upon request.
